# 

**Published:** 1805-08-01

**Authors:** 


### To CORRESPONDENTS.

Dr. Kinglake's Reply to Mr. Mantal, on Gout, will appear in our next Number.

Communications are received from Dr. Thomas, Mr. Harrison, and Mr. Trail.

